# Integrated risk prioritization of rubber additives based on hazard ranking and exposure characterization

**DOI:** 10.1016/j.eehl.2026.100282

**Published:** 2026-09-02

**Authors:** Sihan Wang, Wanting Dai, Bin Wang, Liping Heng

**Affiliations:** a School of Chemistry, Beihang University, Beijing, 100191, China; b State Key Laboratory of Regional Environment and Sustainability, School of Environment, Tsinghua University, Beijing, 100084, China; c Research Institute for Environmental Innovation (Suzhou), Tsinghua University, Suzhou, 215163, China

**Keywords:** Rubber additives, QSAR tools, Risk prioritization, Formulation loading, Multi-model comparison

## Abstract

Rubber products are widely used in transportation, construction, and electronics. At the same time, concern is growing over the environmental and human health risks of rubber additives. However, systematic screening is still limited because few workflows link hazard information with industrial formulation loading data. Here, we developed an integrated high-throughput screening workflow for rubber additives by harmonizing multiple authoritative databases to compile chemical structures, functional information, and representative formulation data. Multi-model and multi-endpoint quantitative structure–activity relationship (QSAR) predictions were integrated using two hazard-ranking strategies, and additional substances of concern were identified using criteria spanning persistence (P), bioaccumulation (B), mobility (M), and toxicity (T). Hazard-based screening identified 82 priority chemicals. For 12 specific rubber additives with publicly accessible formulation ranges, we further incorporated representative formulation loading and a molecular-weight-based migration potential metric. This step produced an exposure factor and an integrated Risk Index (RI). Under exposure-informed conditions, 2,2,4-trimethyl-1,2-dihydroquinoline (TMQ), N-(1,3-dimethylbutyl)-N′-phenyl-p-phenylenediamine (6PPD), and N-isopropyl-N′-phenyl-p-phenylenediamine (IPPD) were identified as top-priority additives by the integrated assessment. Overall, this framework improves the practical relevance of additive prioritization, thereby supporting greener material design, risk management, and substitution assessment in the rubber industry.

## Highlights

• A rubber additive dataset was curated by harmonizing multiple databases.
• Hazard properties were predicted using quantitative structure-activity models.
• Two integration strategies were compared to reduce uncertainty in hazard ranking.
• 82 rubber additives were flagged as priorities using hazard scores.
• The integrated assessment identified TMQ, 6PPD and IPPD as top priority additives.

## Graphical abstract

**Figure:**
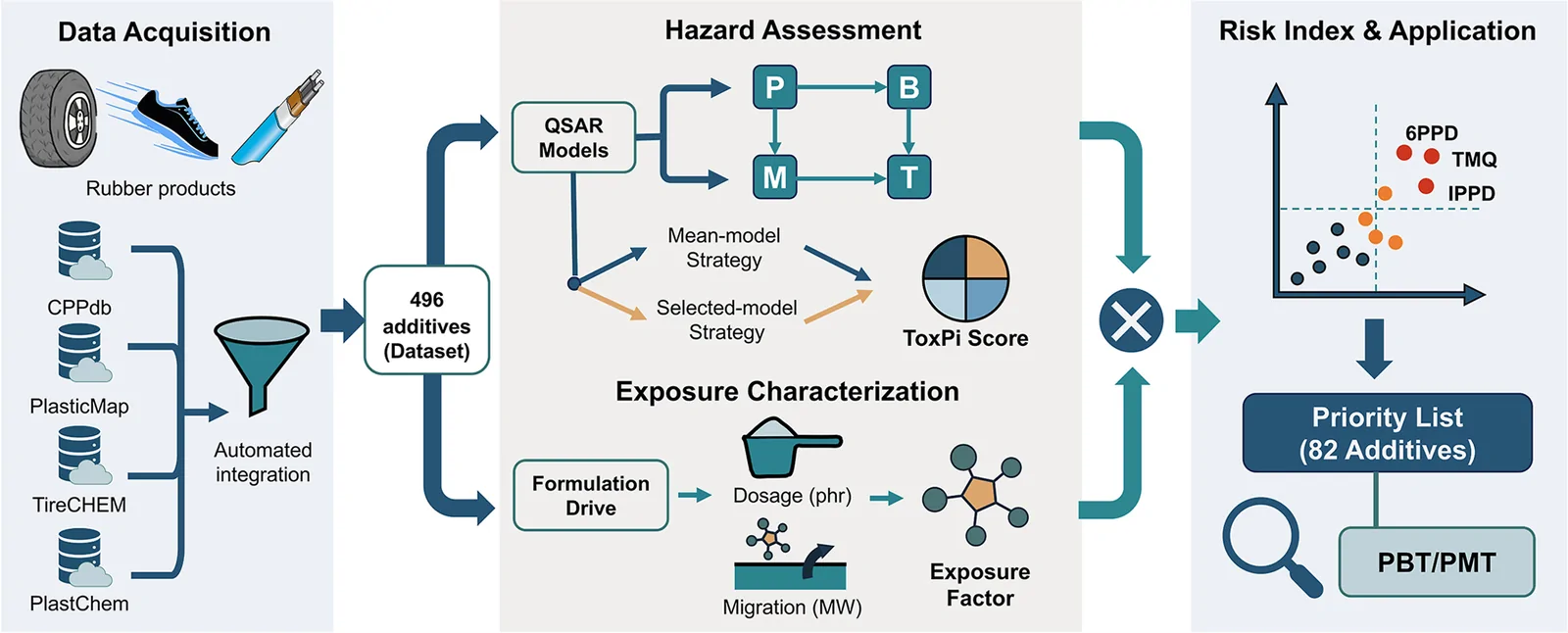

## 1 Introduction

Rubber is widely used in transportation [1], construction [2], electronic devices [3], medical equipment [4], and many other sectors due to its high elasticity, abrasion resistance, and anti-aging properties. To improve processability and extend service life, rubber formulations usually contain multiple additives, including antioxidants, plasticizers, and vulcanizing agents [5]. Many of these additives are not covalently bound to the polymer matrix, so they may migrate and be released during product use or aging, leading to potential ecological and human health risks [6]. With the continued growth in rubber product output, pollution associated with these additives has drawn increasing attention. As a major producer and consumer of rubber additives, China reported a total rubber additive output of 15.879 × 10^5^ t in 2024 [7], accounting for approximately 78% of global production [8]. Among these, vulcanization accelerators accounted for 4.022 × 10^5^ t, antioxidants for 4.516 × 10^5^ t, and processing aids for 3.212 × 10^5^ t [7]. Tires have become a key focus in the study of additive release because of their large-scale use and the generation of wear particles [9]. N-(1,3-dimethylbutyl)-N′-phenyl-p-phenylenediamine (6PPD) and its oxidation product, 6PPD-quinone, have been shown to cause strong acute lethality in aquatic organisms [10]. This finding has drawn international regulatory attention and become a landmark event that has prompted a shift in risk assessment for the rubber industry. Although additive-related pollution has attracted extensive research interest across multiple fields [11], most studies have focused on commodity plastics such as polyethylene [12] and polypropylene [13], while systematic risk screening for rubber additives remains limited. Moreover, current work often centers on a small number of high-profile compounds [14,15] and does not account for differences in real-world formulation loading [16], so screening results may not match practical use conditions.

In chemical management and materials toxicology, persistence (P), bioaccumulation (B), mobility (M), and toxicity (T) are core criteria for evaluating the environmental and human health risks of chemicals [17]. However, experimental evidence for these properties is costly and time-consuming to obtain, and available datasets often have limited coverage. This makes it difficult to support rapid screening across large numbers of chemicals. Therefore, in silico high-throughput screening is widely used as an alternative approach. Quantitative structure–activity relationship (QSAR) models predict the toxicity or environmental impacts of untested chemicals by linking molecular structure to biological activity or toxicological effects [18]. This enables rapid, cost-effective preliminary screening for many chemicals without additional animal testing. Because models differ in training data, algorithms, and applicability domains, their predictions can vary and carry uncertainty. If a single model is chosen at random, the results may be biased. Therefore, more robust ensemble strategies are needed to support reliable chemical prioritization.

To address these gaps, we developed a systematic risk assessment framework for rubber-related applications. First, an automated data processing workflow was used to integrate multiple authoritative databases through cross-database extraction, identifier checking, and deduplication, building an additive dataset with structural identifiers and functional labels. Next, multi-model and multi-endpoint in silico predictions were performed for the P, B, M, and T properties. In parallel, two complementary integration strategies were applied to reduce uncertainty. We further used the Toxicological Priority Index (ToxPi) to standardize and integrate predicted endpoint values, producing a hazard ranking on a unified scale. This ranking was used to support preliminary screening and to guide follow-up actions. On this basis, representative formulation loading, defined as the midpoint of the reported formulation range for each additive, was introduced to characterize differences in typical use levels. We also used a molecular-weight-based relative diffusion coefficient to represent release potential. These components were combined to build an exposure factor, which was coupled with hazard intensity to derive an integrated risk index (RI). This step transforms hazard-based prioritization into a risk ranking that better reflects differences in typical additive use levels and relative release potential under practical application conditions. Overall, the workflow links automated data curation, control of model uncertainty, and formulation-informed exposure adjustment. It provides data support for priority lists of rubber additives, alternative assessment, and green design, and helps balance performance improvement with environmental protection.

## 2 Materials and methods

### 2.1 Chemical selection and data processing

Information on rubber-related chemicals was obtained from four databases hosted on the NORMAN platform (https://www.norman-network.com/nds/susdat/), including the database of chemicals associated with plastic packaging [19] (CPPdb List A and List B), the global market database of plastic monomers, additives, and processing aids [20] (PlasticMAP), the tire-related chemicals database [21] (TireCHEM), and the PlastChem database [22]. Candidate chemicals were selected using three criteria. (1) The database record had to explicitly indicate that the chemical is used in rubber manufacturing or has been detected in rubber products. (2) The chemical had to have a defined functional category. (3) The chemical had to have a unique identifier, with at least a CAS number or a Simplified Molecular Input Line Entry System (SMILES) string provided. Notably, as non-intentionally added substances (NIAS) generally lack stable intentional inclusion levels and are more strongly influenced by production conditions, aging, and environmental transformation, this study focused on intentionally added substances relevant to source-oriented formulation control. Nevertheless, due to limitations in the underlying database, a small number of NIAS in actual rubber systems may still have been unavoidably retained in the broader dataset.

We then applied an automated data extraction and processing workflow to harmonize and merge rubber-related chemicals across the four databases. The Python code used for batch processing is provided in Fig. S1. Key fields were collected, including chemical name, CAS number, molecular formula, functional category, and SMILES string. Duplicate records were merged, and entries that were invalid, incomplete, or lacked clear functional information were removed. This procedure initially yielded 638 rubber-related chemicals that met the inclusion criteria. For chemicals with a CAS number but without a SMILES string, structural information was further supplemented using PubChem (https://pubchem.ncbi.nlm.nih.gov/). 33 chemicals were excluded because reliable structural information could not be obtained. Considering the applicability domain limitations of the QSAR tools [23], 109 additional chemicals with SMILES strings did not return valid endpoint predictions and were excluded from subsequent integrated analyses. These substances mainly fell outside the QSAR-applicable chemical space of the present framework, including substances of unknown or variable composition, complex reaction products, or biological materials (UVCBs), polymers, most inorganic substances, organometallic compounds, and chemicals lacking useable prediction data. Ultimately, 496 additives were retained as the candidate set for hazard assessment and risk prioritization.

### 2.2 QSAR tools and endpoint selection

Hazard endpoint values for rubber additives were predicted using widely used computational toxicology and QSAR platforms [16,18,24]. These values were obtained from 45 QSAR models, including models from open-access tools such as EPI Suite, VEGA [25], TEST [26], OPERA, and OCHEM [27]. Models from the U.S. Environmental Protection Agency’s (EPA) Collaborative Estrogen Receptor Activity Prediction Project (CERAPP) and Collaborative Modeling Project for Androgen Receptor Activity (CoMPARA) were also included [28]. Together, these tools cover physicochemical properties, environmental fate-related descriptors, general toxicity endpoints, and receptor-based bioactivity predictions, and have been widely used in chemical screening, hazard assessment, and prioritization studies. These models covered 16 hazard endpoints across the P, B, M, and T properties. The toxicity properties included aquatic ecotoxicity, endocrine-disrupting effects, mutagenicity, carcinogenicity, and developmental toxicity. In addition, when a tool provided both experimental data and predicted values for the same endpoint, experimental data were preferentially used to improve the reliability of endpoint assignment. Detailed information on each endpoint, data sources, and the corresponding software and models is provided in Table S1. When multiple predictions were available for the same endpoint, discrepancies among models were handled using the endpoint integration strategies described in Section 2.3.

#### 2.2.1 Criterion 1: persistence (P)

Persistence reflects the extent to which a chemical resists degradation in environmental media and is commonly assessed by biodegradation rates or degradation half-lives. In this study, biodegradability was predicted using the BIOWIN models in EPI Suite. Specifically, BIOWIN 2 and BIOWIN 6 were used to estimate the probability of biodegradation under aerobic and anaerobic conditions, respectively, and BIOWIN 3 was used to estimate the time frame for ultimate biodegradation. A chemical was classified as persistent when BIOWIN 2 or BIOWIN 6 indicated low biodegradability (value < 0.5) and BIOWIN 3 predicted an ultimate biodegradation time frame longer than several months (value < 2.25) [29].

#### 2.2.2 Criterion 2: bioaccumulation (B)

Bioaccumulation describes the extent to which a chemical accumulates in organisms and is commonly quantified using the bioconcentration factor (BCF). Higher BCF values indicate a greater potential for enrichment in aquatic biota. In this study, log BCF values were predicted using the BCFBAF model in EPI Suite together with multiple machine learning and statistical models available on the VEGA platform. A chemical was classified as bioaccumulative (B) when BCF exceeded 2000 (log BCF > 3.3), and as very bioaccumulative (vB) when BCF exceeded 5000 (log BCF > 3.7) [30].

#### 2.2.3 Criterion 3: mobility (M)

Mobility describes the ability of a chemical to migrate within environmental media, especially to disperse and be transported over long distances in aquatic systems. Highly mobile substances can spread rapidly across regions, thereby increasing the spatial extent of exposure and associated risks [31]. In mobility assessment, the soil organic carbon–water partition coefficient (*K*_oc_) is widely used. Lower *K*_oc_ values suggest a higher tendency for a chemical to enter water and migrate. In this study, log *K*_oc_ values were predicted using the KOCWIN model in EPI Suite and the OPERA software. Classification followed the criteria proposed by Santos and Nardocci et al. [32]. Under environmental pH conditions between 4 and 9, a chemical was classified as mobile (M) when log *K*_oc_ was below 4, and as very mobile (vM) when log *K*_oc_ was below 3.

#### 2.2.4 Criterion 4: toxicity (T)

Toxicity is a key dimension in environmental and human health risk assessment and includes aquatic ecotoxicity, carcinogenicity, mutagenicity, developmental toxicity, and potential endocrine-disrupting effects. Following the criteria for identifying persistent, bioaccumulative, and toxic (PBT) and persistent, mobile, and toxic (PMT) substances in REACH Annex XIII [33], multiple QSAR tools, including VEGA, TEST, OCHEM, and OPERA, were used to cover 11 toxicity-related endpoints and to characterize chemical toxicity. A chemical was classified as toxic when it met any of the following conditions [17]. (1) Aquatic ecotoxicity. Chronic toxicity met the criterion of no observed effect concentration (NOEC) ≤ 1 mg/L, or acute toxicity met the criterion of EC50 or LC50 ≤ 1 mg/L. In addition, when BCF exceeded 50, acute toxicity also met the criterion of EC50 or LC50 ≤ 10 mg/L (2) Carcinogenicity. The outcome was predicted to be positive by VEGA. (3) Mutagenicity. The outcome was predicted to be positive by VEGA. (4) Developmental toxicity. The developmental toxicity score predicted by TEST exceeded 0.5, or VEGA classified the endpoint as positive. (5) Endocrine-disrupting activity. Models predicted positive binding, agonist, or antagonist activity for the estrogen receptor (ER) or androgen receptor (AR), or predicted interference with thyroid receptor (TR) binding.

### 2.3 Endpoint integration and ToxPi-based hazard scoring

To reduce the impact of bias from any single model on prioritization, two processing strategies were applied to multi-model predictions for the same endpoint, and their ranking consistency was compared to evaluate result robustness. The first strategy was the mean-model approach, in which predictions from multiple models for a given endpoint were aggregated as the arithmetic mean [34]. The second strategy was the selected-model approach, which was applied as an endpoint-specific model comparison and integration procedure rather than a model-training framework. For endpoints with sufficient experimental support and at least three available model predictions, model outputs were compared against harmonized experimental values compiled from software-associated databases, and the relatively better-performing model was selected for subsequent integration. Continuous and categorical endpoints were handled separately according to their data structure. Endpoints lacking corresponding experimental values or having insufficient experimental records were not included in the endpoint-specific model selection. Detailed descriptions of the experimental data support, endpoint-specific evaluation procedures, and performance metrics are provided in Text S1.

For categorical endpoints, model outputs were harmonized as 1 for inactive, 2 for uncertain, and 3 for active. Under the mean-model approach, the arithmetic mean of the recoded values was converted back to endpoint classes using 1.0 ≤ *x* < 1.5 for inactive, 1.5 ≤ *x* < 2.5 for uncertain, and 2.5 ≤ *x* ≤ 3.0 for active. Under the selected-model approach, the class predicted by the best-performing model was used directly. Final P, B, M, and T classifications were then determined from the integrated endpoint results under each strategy, rather than by automatically taking the most conservative output from individual models.

To further evaluate the robustness of the prioritization results, a sensitivity analysis was performed by comparing the mean-model, selected-model, best-case, and worst-case scenarios. Ranking stability was assessed using Spearman rank correlation (*ρ*), top 10% overlap, and retention of the top 10% within the top 15% tier. Details are provided in Text S2 and Tables S2 and S3.

The two strategies described above produced standardized hazard predictions across multiple endpoints. These results were further combined using the ToxPi method. ToxPi rescales results from different hazard endpoints to a comparable scale and aggregates them through weighted summation under predefined weights, thereby generating an overall hazard score for each chemical [32]. The overall score was calculated according to Eq. (1).

(1) $$Tox{Pi}_{i}=\sum_{j=1}^{n}\left(\omega_{j}\times S_{i,j}\right)$$

Where, *S*_*i,j*_ represents the standardized score of chemical *i* for hazard endpoint *j*; ω_*j*_ denotes the weight assigned to endpoint *j*, and *n* refers to the total number of hazard endpoints included in the integration. The weights assigned to each endpoint are listed in Table S1.

Finally, the mean-model approach and selected-model approach generated their respective ToxPi composite scores, which were used to compare the consistency and differences in chemical prioritization. Chemicals with higher ToxPi scores and higher ranks were considered to have greater overall hazard potential and should be prioritized for further toxicity verification and risk management.

### 2.4 Exposure proxy and risk index construction

In risk assessment, hazard reflects the potential adverse effects of a chemical, whereas actual risk also depends on the level of exposure under realistic use conditions. To make the prioritization of rubber additives more relevant to formulation-based application conditions, an exposure factor was introduced to the hazard-based ToxPi overall score. This exposure factor was designed as a generic, source-oriented proxy for comparative prioritization, rather than a receptor-specific exposure estimate for workers or the general population. An integrated RI was then constructed for relative risk prioritization [24]. The workflow consisted of three steps. First, the hazard score was calculated by integrating multi-endpoint QSAR results within the ToxPi framework. Second, exposure was estimated using typical formulation loading and migration potential. Third, hazard and exposure were coupled to derive the RI [35].

The exposure component was represented by a formulation-loading proxy to characterize the typical use level of additives in rubber formulations. Based on rubber handbooks [36,37] and related studies [38], representative additives from several functional categories commonly used in basic rubber formulations were compiled for different product types, including tires, shoe soles, hoses, and vibration-damping components (Table S4). These data were used to document typical formulation practice and data availability. Among the listed additives, only 12 specific chemicals were ultimately retained for the exposure and RI analyses, because they had publicly accessible formulation ranges and were compatible with the present concentration-migration framework. For each chemical, the midpoint of the reported formulation range was used as its representative formulation loading, expressed in parts per hundred rubber (phr), i.e., the mass of additive per 100 parts by weight of rubber. This metric represents the typical formulation-level use of an additive rather than its absolute concentration in the final product.

Migration potential was estimated using the empirical relationship for the upper limit diffusion coefficient of additives in polymers provided by the Piringer interaction model [39]. Under the relative screening framework used here, target chemicals were compared under the assumption of the same rubber matrix type and ambient temperature (T = 298.15 K). Accordingly, matrix-dependent parameters and temperature-related constants were treated as fixed, and only the molecular-weight-dependent term was retained to define the relative diffusion coefficient (*D*_*rel,i*_). This simplified proxy is mainly applicable to intact, unaged rubber matrices under comparable conditions, assuming that the restrictive effects of crosslink density and solid fillers are relatively uniform across the screened additives [40,41]. However, in scenarios dominated by non-diffusive mechanisms, such as abrasion-driven release [42], severe matrix aging or surface cracking [40], medium-induced swelling, or oil-assisted transport [43], this diffusion-based proxy may not be valid or dominant. The formulation loading proxy (*c*_*i*_) was multiplied by *D*_*rel,i*_ to obtain the raw exposure indicator (*EI*_0,*i*_). Because the diffusion term varies exponentially, the exposure baseline values can span multiple orders of magnitude. We therefore applied a logarithmic transformation to *EI*_0,*i*_ to improve numerical stability and to reduce the dominance of extreme values on ranking. The exposure metric was then min–max normalized across the study set, scaling the exposure factor to the range from 0 to 1. This ensured that the exposure factor was on the same scale as the ToxPi score, thereby facilitating construction of the integrated risk index. The equations are given below.

(2) $$c_{i}={phr}_{i}$$

(3) $$D_{rel,i}=\exp\left({{-0.1351MW}_{i}}^{2/3}+0.003MW_{i}\right)$$

(4) $${EI}_{0,i}=c_{i}\cdot D_{rel,i}$$

(5) $${Exposure}_{i}=\log_{10}\left({EI}_{0,i}\right)=\log_{10}\left(c_{i}\right)+\log_{10}\left(D_{rel,i}\right)$$

(6) $${Exposure}_{normalized,i}=\frac{{Exposure}_{i}-{Exposure}_{\min}}{{Exposure}_{\max}-{Exposure}_{\min}}$$

(7) $${RI}_{i}={ToxPi}_{i}\times {Exposure}_{normalized,i}$$

Here, *i* denotes additive *i*; *c*_*i*_ denotes the formulation loading proxy of additive *i*; *MW*_*i*_ is the relative molecular weight of additive *i*; *D*_*rel,i*_ is the relative diffusion coefficient, representing differences in migration potential driven by molecular weight under the same rubber matrix and temperature assumptions; *EI*_0,*i*_ is the raw exposure value; *Exposure*_*i*_ is the log-transformed exposure metric; *Exposure*_*normalized,i*_ is the normalized exposure factor, where *Exposure*_*min*_ and *Exposure*_*max*_ are the minimum and maximum values within the study set, respectively; *ToxPi*_*i*_ is the composite hazard score; *RI*_*i*_ is the integrated risk index, where higher values indicate higher relative risk priority. To evaluate whether the prioritization was sensitive to the coupling form, we additionally compared the multiplicative RI with an additive weighted-sum alternative, and the results are summarized in Table S5.

## 3 Results and discussion

### 3.1 Selection of best-performing models

**Fig. 1:**
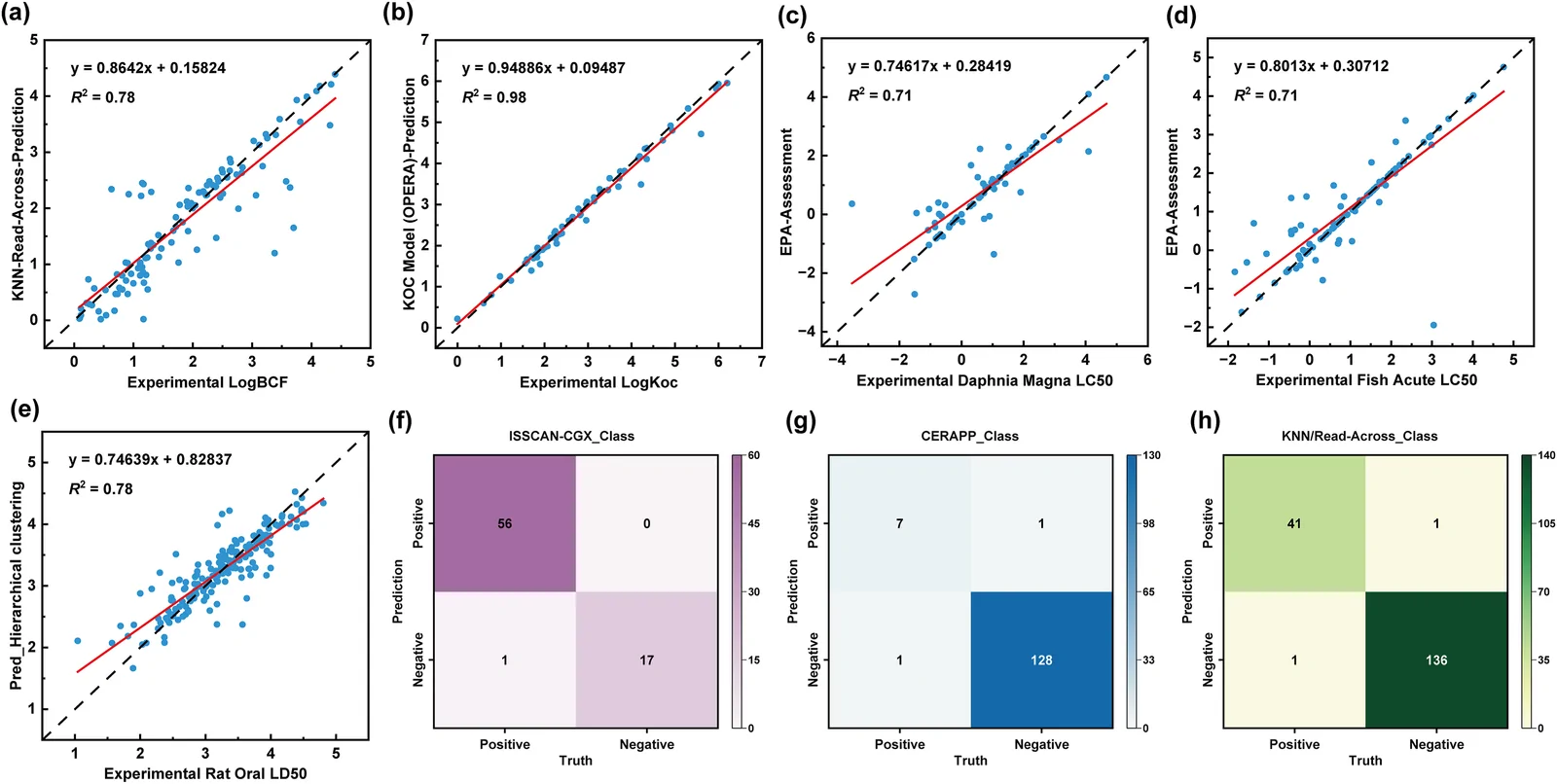
Model performance for endpoints with experimental reference data. Quantitative endpoints (a–e) are shown as predicted versus experimental values, and qualitative endpoints (f–h) are shown as confusion matrices. (a) BCF. (b) *K*_oc_. (c) Acute *Daphnia magna* toxicity LC50. (d) Acute fish toxicity LC50. (e) Oral rat toxicity LD50. (f) Carcinogenicity. (g) ER binding activity. (h) Mutagenicity. In panels (a–e), points represent individual chemicals, the red line is the linear regression fit, and the dashed line indicates *y* = *x*. In panels (f–h), the x axis shows the true class and the y axis shows the predicted class, with cell values showing sample counts. BCF, bioconcentration factor; *K*_oc_, organic carbon–water partition coefficient; LC50, median lethal concentration; LD50, median lethal dose; EPA, U.S. Environmental Protection Agency.

Fig. 1 summarizes the prediction versus experimental fits for continuous endpoints (Fig. 1a–e) and the confusion matrices for categorical endpoints (Fig. 1f–h). For continuous endpoints, the best-performing model for the bioaccumulation metric log BCF was the KNN-Read-Across model on the VEGA platform, with an *R*^2^ of 0.78 (Fig. 1a), indicating that the model explained approximately 78% of the variance in the data. For mobility, the OPERA *K*_oc_ model provided the best fit, with *R*^2^ = 0.98 and a regression slope close to 1 (Fig. 1b), suggesting strong agreement between predictions and experimental values with limited systematic bias. For the fish acute LC50, rat oral LD50, and *Daphnia* magna LC50 endpoints, the VEGA EPA model performed best, with *R*^2^ values of about 0.70, also indicating high predictive accuracy. Overall, the selected models for continuous endpoint values ranged from *R*^2^ = 0.71 to 0.98, supporting their reliability. The fitting results for the remaining candidate models are provided in Figs. S2–S6.

For categorical endpoints, the best-performing model for the carcinogenicity endpoint was the Carcinogenicity model (IRFMN-ISSCAN-CGX), with balanced accuracy (BA) = 0.991, Matthews correlation coefficient (MCC) = 0.963, and recall = 0.983 (Fig. 1f). As indicated by the confusion matrix, this model produced no false positives and only one chemical showed a discrepancy between the prediction and the experimental result, demonstrating strong identification of positive cases in this dataset. For ER binding activity, the IRFMN-CERAPP model performed best, with BA of 0.934, MCC of 0.867, and recall of 0.875 (Fig. 1g). This model distinguished negative samples accurately, with a false positive rate below 1%, while showing limited false negatives, with one missed positive among eight positive samples. For mutagenicity, the best-performing model was the Mutagenicity (Ames test) model (KNN-Read-Across) on the VEGA platform, with BA of 0.984, MCC of 0.969, and recall of 0.976 (Fig. 1h). Compared with experimental results, the overall agreement of this model was close to 99%. It missed 1 positive among 42 positive samples (approximately 2.4%) and misclassified 1 negative among 137 negative samples (approximately 0.7%), indicating stable performance for both classes. Confusion matrices and performance metrics for the remaining candidate models are provided in Figs. S7–S9.

### 3.2 ToxPi-based hazard prioritization

Using endpoint predictions generated by the two approaches, ToxPi-based hazard ranking was performed for 496 rubber additives. The overall results are shown in Fig. S10. The two ranking curves overlapped closely, indicating strong agreement between the approaches in overall prioritization. The distribution of ToxPi scores showed a pronounced long-tail pattern. Most chemicals had ToxPi values between 0.30 and 0.60, whereas only a small fraction fell into the high-score range. The approximately 50 top-ranked chemicals exhibited relatively higher overall hazard potential. Accordingly, chemicals ranked in the top 10% were classified as high hazard, those ranked from 10% to 50% were classified as medium hazard, and those ranked in the bottom 50% were classified as low hazard.

A comparison of the high-hazard chemicals identified by the two approaches (Table 1) showed that the ranking positions of most chemicals were broadly consistent, and this pattern remained when the analysis was extended to all 496 chemicals. Only a limited number of chemicals shifted near the category boundaries under the selected-model approach, with 10 chemicals moving from the high-hazard category to the medium-hazard category and 10 shifting in the opposite direction. These differences mainly arose from two sources. First, aquatic toxicity endpoints showed substantial model-to-model variation. Because QSAR models differ in training data composition, applicability domains, and treatment of ionization states and solubility constraints, predicted LC50 and EC50 values for the same chemical can differ by 10^2^ to 10^4^, and this uncertainty could remain appreciable after log transformation. Second, categorical endpoints such as carcinogenicity, mutagenicity, and receptor binding activity showed label discordance across models. After normalization and weighted aggregation in the ToxPi framework, such endpoint disagreements could lead to rank adjustments for a small number of chemicals near the category boundaries. Sensitivity analysis across the four scenarios showed that the overall ranking remained highly consistent, with *ρ* values ranging from 0.846 to 0.957. The overlap of the top 10% chemicals between the two routine approaches was 78.0%, and most chemicals ranked in the top 10% under the mean-model or selected-model approach remained within the top 15% even under the best-case and worst-case scenarios (Table S3). Overall, these findings indicate that model-related uncertainty mainly affected local ordering near the top 10% cut-off, whereas the identification of the core high-risk chemicals remained stable.

**Table 1:** Comparison of high priority chemicals identified by the two approaches.

Name	CAS No.	Cat.	Rank	
			Mean	Selected
2,2′,3,3′,4-Pentachlorobiphenyl	11097-69-1	N	1	1
2,2′,4,4′-Tetrachlorobiphenyl	53469-21-9	N	2	2
Hexachlorobenzene	118-74-1	N	3	6
Decabromodiphenyl oxide	1163-19-5	I	4	9
Mirex	2385-85-5	U	5	4
Pentachlorothiophenol	133-49-3	I	6	3
(Pentabromophenyl)methyl acrylate	59447-57-3	I	7	7
Benzo[k]fluoranthene	207-08-9	N	8	17
7,8-Benzfluoranthene	205-82-3	N	9	15
Indeno[1,2,3-cd]pyrene	193-39-5	N	10	14
Benzo[b]fluoranthene	205-99-2	N	11	16
2,3,4,6,7,8-Hexachlorodecane	108171-26-2	U	12	57
Pentachlorobenzene	608-93-5	N	13	12
Bis(2,4-dichlorobenzoyl) peroxide	133-14-2	I	14	8
2,4,6-Tri-tert-butylphenol	732-26-3	I	15	13
2-(2H-Benzo[d][1,2,3]triazol-2-yl)-4,6-di-tert-butylphenol	3846-71-7	I	16	11
N,N′-Di-2-naphthyl-p-phenylenediamine	93-46-9	I	17	29
1,2,5,6,9,10-Hexabromocyclododecane	3194-55-6	I	18	19
γ-Hexabromocyclododecane	134237-52-8	I	19	20
C10–C13 chlorinated paraffins	85535-84-8	I	20	64
4,4′-Methylenebis(2-chloroaniline)	101-14-4	U	21	26
N,N′-Ditolyl-p-phenylenediamine	27417-40-9	I	22	25
Benzo[e]pyrene	192-97-2	N	23	36
2,4-Di-tert-butyl-6-(5-chloro-2H-benzotriazol-2-yl)phenol	3864-99-1	I	24	21
Tetrabromobisphenol A	79-94-7	I	25	24
Octamethylcyclotetrasiloxane	556-67-2	N	26	23
N,N′-Bis(1,4-dimethylpentyl)-P-phenylenediamine	3081-14-9	I	27	50
N,N′-Diphenyl-p-phenylenediamine	74-31-7	I	28	31
Benzo[a]pyrene	50-32-8	N	29	28
N-Cyclohexyl-N′-phenyl-4-phenylenediamine	101-87-1	I	30	59
Mercury, (neodecanoato-kappaO)phenyl-	26545-49-3	U	31	119
UV-328	25973-55-1	I	32	34
3,3′-Dichlorobenzidine	91-94-1	U	33	33
Tetrabromophthalic anhydride	632-79-1	I	34	18
2,6-Di-tert-butyl-4-ethylphenol	4130-42-1	I	35	30
Benz(a)anthracene	56-55-3	N	36	35
Perylene	198-55-0	N	37	38
4-tert-Octylphenol	140-66-9	U	38	32
Acridine, 9,10-dihydro-9,9-dimethyl-	6267-02-3	N	39	55
1,4-Benzenediamine, N-(1,3-dimethylbutyl)-N′-phenyl-	793-24-8	I	40	58
2,3-Dimethyl-2,3-diphenylbutane	1889-67-4	U	41	56
Kathon 930	64359-81-5	U	42	63
10,10′-Oxybisphenoxarsine	58-36-6	I	43	27
Pyrene	129-00-0	N	44	43
Chrysene	218-01-9	N	45	42
Phenol, 2-(1,1,3,3-tetramethylbutyl)-	27193-28-8	U	46	54
Triclosan	3380-34-5	U	47	41
N,N-Ethylene-bis(tetrabromophthalimide)	32588-76-4	I	48	60
Octrizole	3147-75-9	I	49	48
Tributyltin oxide	56-35-9	U	50	80
2-(2H-Benzotriazol-2-yl)-4-(tert-butyl)-6-(sec-butyl)phenol	36437-37-3	I	51	37
3,3′-Dimethylbenzidine	119-93-7	U	53	49
2,2-Bis[3,5-dibromo-4-(2,3-dibromopropoxy)phenyl]propane	21850-44-2	I	56	5
1,1,2,2,3,3-Hexabromocyclododecane	25637-99-4	I	57	40
2,6-di-tert-Butyl-4-(dimethylaminomethyl)phenol	88-27-7	I	61	22
N-Isopropyl-N′-phenyl-p-phenylenediamine	101-72-4	I	63	39
N-Phenyl-1-naphthylamine	90-30-2	I	64	46
Bis[4-(2-phenyl-2-propyl)phenyl]amine	10081-67-1	I	71	44
1,1'-(Isopropylidene)bis(3,5-dibromo-4-(2,3-dibromo-2-methylpropoxy)benzene)	97416-84-7	I	77	10
Phenol, 2,6-bis((3-(1,1-dimethylethyl)-2-hydroxy-5-methylphenyl)octahydro-4,7-methano-1H-indenyl)-4-methyl-	31851-03-3	I	157	47

Cat. indicates the putative source category of each chemical in rubber-related materials: I, intentional additive; N, mainly detected contaminant/impurity (non-intentional); U, uncertain. I refers to chemicals with defined technical functions in rubber formulations. N refers to chemicals more likely to occur as impurities, by-products, legacy pollutants, or detected contaminants in rubber products, rather than as intentionally added additives. U indicates that the source status of a chemical in rubber products could not be assigned with sufficient confidence based on the available information.

Taking C10–C13 chlorinated paraffins (CAS No. 85535-84-8) as an example, this substance can be used in rubber as a plasticizer or flame retardant and is classified as short-chain chlorinated paraffins (SCCPs). SCCPs have attracted long-term international concern because of their environmental persistence, high potential for long-range transport, toxicity to biota, and bioaccumulation [44]. SCCPs have been identified as substances of great concern by the European Chemicals Agency, selected by the U.S. EPA for action plan development under the Toxic Substances Control Act, and subsequently listed in Annex A to the Stockholm Convention in 2017 [[45], [46], [47]]. In China, the List of New Pollutants for Priority Control (2023 Edition) has prohibited the production and processing use of SCCPs. Notably, this substance was classified as high hazard only under the mean-model approach. This finding suggests that prioritization based on a single best-performing model may underestimate high-profile substances for which regulatory and toxicological evidence is highly consistent. A similar pattern was observed for tributyltin oxide (TBTO, CAS No. 56-35-9). TBTO was ranked within the top 50 under the mean-model approach but shifted to a lower rank under the selected-model approach and was not assigned to the high-priority category. Previous studies have shown that tributyltin compounds (TBT) exhibit pronounced environmental persistence. They can strongly bind to suspended matter and sediments and be released gradually from these substrates over extended periods, up to 30 years. TBT is also recognized as an endocrine-disrupting chemical and shows extremely high aquatic toxicity. TBT exposure has been reported to alter sex ratios [48], induce sperm abnormalities [49], and disrupt reproductive behavior [50] in aquatic species. It can also cause developmental abnormalities and increased mortality in mollusks, even at concentrations as low as ng/L [51]. In fish, TBTO exposure has been associated with marked thymic atrophy and impaired immune function in European flounder [52]. The International Maritime Organization (IMO) has prohibited the use of TBT-based antifouling agents and biocides. These chemicals are also listed in Annex III of the Rotterdam Convention and are subject to the prior informed consent (PIC) procedure. Collectively, these results indicate that incorporating multi-model consensus at the hazard screening stage can improve alignment with regulatory practice and reduce ranking shifts driven by bias from a single model.

However, the selected-model approach is not redundant and provides complementary value. When relatively sufficient experimental reference data are available for specific endpoints, this approach can highlight potential high-hazard signals that may be attenuated by averaging across models. For example, N-isopropyl-N′-phenyl-1,4-phenylenediamine (IPPD, CAS No. 101-72-4) is a key antiozonant in the rubber industry. Its surface migration and release during tire wear can promote environmental emissions, and it can enter urban runoff and sediments via particulate matter [53]. Experimental evidence has shown that IPPD is a strong human sensitizer and exhibits high toxicity to aquatic organisms with persistent effects [54]. Further studies have reported that its oxidation product, IPPD-quinone (IPPD-Q), and chlorinated by-products formed during drinking water disinfection can be more embryolethal than the parent compound, posing a less apparent threat to drinking water safety [55]. These findings indicate that, although IPPD has not been listed as a substance of very high concern (SVHC), its complex secondary risks and characteristic migration behavior have raised substantial scientific concern. In Table 1, the rank of IPPD increased under the selected-model approach. This shift suggests that when endpoint-specific models show strong performance and closer agreement with experimental observations, selecting the best-performing model can amplify high-hazard signals and improve sensitivity for identifying potentially hazardous substances. A similar pattern was observed for 2-(2H-benzotriazol-2-yl)-4-(tert-butyl)-6-(sec-butyl)phenol (UV-350, CAS No. 36437-37-3), a benzotriazole ultraviolet absorber commonly used in rubber. Toxicological concern for UV-350 is mainly associated with liver and kidney target-organ effects under repeated exposure, indicating potential systemic toxicity under long-term and cumulative exposure scenarios [56]. The Annex XV identification dossier for SVHC in the European Union classifies UV-350 as very persistent and very bioaccumulative (vPvB). Reported log *K*_ow_ values are approximately 6.3 to 7.1, and fish BCF values can reach 7700 to 13,000. These properties indicate low biodegradability and slow degradation in environmental media. In addition, strong sorption and low mobility in the aqueous phase, reflected by high *K*_oc_ values, suggest a tendency to partition into particles and sediments and to accumulate along food webs. Moreover, following a screening assessment released in 2024, the European Chemicals Agency (ECHA) raised concerns about initiating restrictions on the content of benzotriazoles such as UV-350 in articles. Together, these lines of evidence suggest that the selected-model approach can offset the conservativeness of the mean-model approach and facilitate identification of additional chemicals with potentially high-hazard profiles.

Building on the above comparison of rank differences for selected high-profile substances, we further extended the analysis to a class of contaminants that ranked consistently high in this study, namely polycyclic aromatic hydrocarbons (PAHs). We identified 13 PAHs in total (Table S6), of which 10 were classified as high hazard substances. PAHs are not functional rubber additives in the conventional sense. Instead, they are repeatedly detected as impurities or byproducts throughout the life cycle of plastic and rubber materials [57]. In rubber systems, PAHs mainly originate from rubber process oils and carbon black. The former may contain different levels of PAHs across batches, while the latter may introduce PAH contamination during the incomplete combustion of feedstock oils under high-temperature pyrolysis conditions [58]. Therefore, PAHs should be regarded primarily as detected contaminants/impurities among rubber-related chemicals, rather than as direct functional formulation additives. Although they are not intentionally added, they often rank high in hazard screening and have received broad attention, and thus remain important for pollution identification, risk warning, and source control. However, they should not be interpreted in the same way as intentionally added rubber additives in the context of formulation substitution or green design.

From a toxicological perspective, PAHs can exhibit endocrine-disrupting activity and have carcinogenic, teratogenic, and mutagenic properties. They are also associated with adverse cardiovascular and respiratory outcomes [59]. Given these hazard profiles, Rich PAH process oils have been strictly restricted and are being progressively replaced by low-PAH mineral oils or synthetic esters. In recent years, the use of more environmentally benign alternatives, such as vegetable oils or animal fats, has also been encouraged as plasticizing oils [60]. From a regulatory perspective, Regulation (EC) No 1907/2006, Registration, Evaluation, Authorisation and Restriction of Chemicals (REACH), Annex XVII, Entry 50 sets concentration limits for eight highly carcinogenic PAHs in extender oils placed on the market for the manufacture of tires and tire components. Specifically, benzo[a]pyrene (BaP) must not exceed 1 mg/kg, and the sum of the eight PAHs must not exceed 10 mg/kg. The restriction was subsequently extended to general consumer products. For rubber components that can come into direct, prolonged, or repetitive short-term contact with skin or the oral cavity, each of the eight PAHs must not exceed 1 mg/kg, and the limit is 0.5 mg/kg for toys and childcare articles. In China, carbon black used as an additive in food-contact materials and articles, including rubber, is also subject to control requirements. For example, GB 9685–2016 specifies that the toluene extractables of carbon black must be below 1% and that BaP must be below 0.25 mg/kg.

Notably, among the eight PAHs subject to strict regulatory limits, seven were classified as high hazard substances in this study. This result indicates consistency between our hazard-based prioritization and regulatory concern. Overall, PAHs tended to rank near the top in our framework, which is consistent with their well-established hazard profiles. However, hazard priority should be distinguished from actual risk in product-specific contexts. Because regulations impose stringent limits on PAH concentrations in rubber products, exposure levels in compliant products are often substantially constrained. Previous risk assessment studies have reported no substantial risk associated with consumer products made from recycled rubber. Under the dominant exposure pathway, typically inhalation, the associated risks are generally considered acceptable [61]. This contrast further underscores the need to incorporate exposure proxies, such as formulation loading, in addition to hazard ranking when constructing integrated risk prioritization, in order to support risk judgments that better reflect real-world application scenarios.

### 3.3 Identification of PBT and PMT substances

Within the REACH regulatory framework, substances that meet the criteria for persistent, bioaccumulative and toxic (PBT) or vPvB can be identified as SVHC. This designation may trigger further assessment and risk management actions. Similarly, persistent, mobile, and toxic (PMT) or very persistent and very mobile (vPvM) substances are considered to warrant a comparable level of concern because they may pose long-term threats to drinking water safety [62]. The main difference between these groups lies in their dominant exposure pathways. PBT/vPvB substances are mainly associated with bioaccumulation and food-web magnification. In contrast, PMT/vPvM substances are more relevant to persistence and transport in aquatic environments [63]. For both groups, adverse effects may persist over long time scales and are difficult to reverse once emissions occur. Therefore, in addition to the top 50 chemicals identified by the overall hazard ranking, substances meeting the PBT or PMT criteria were also included in the priority list.

**Fig. 2:**
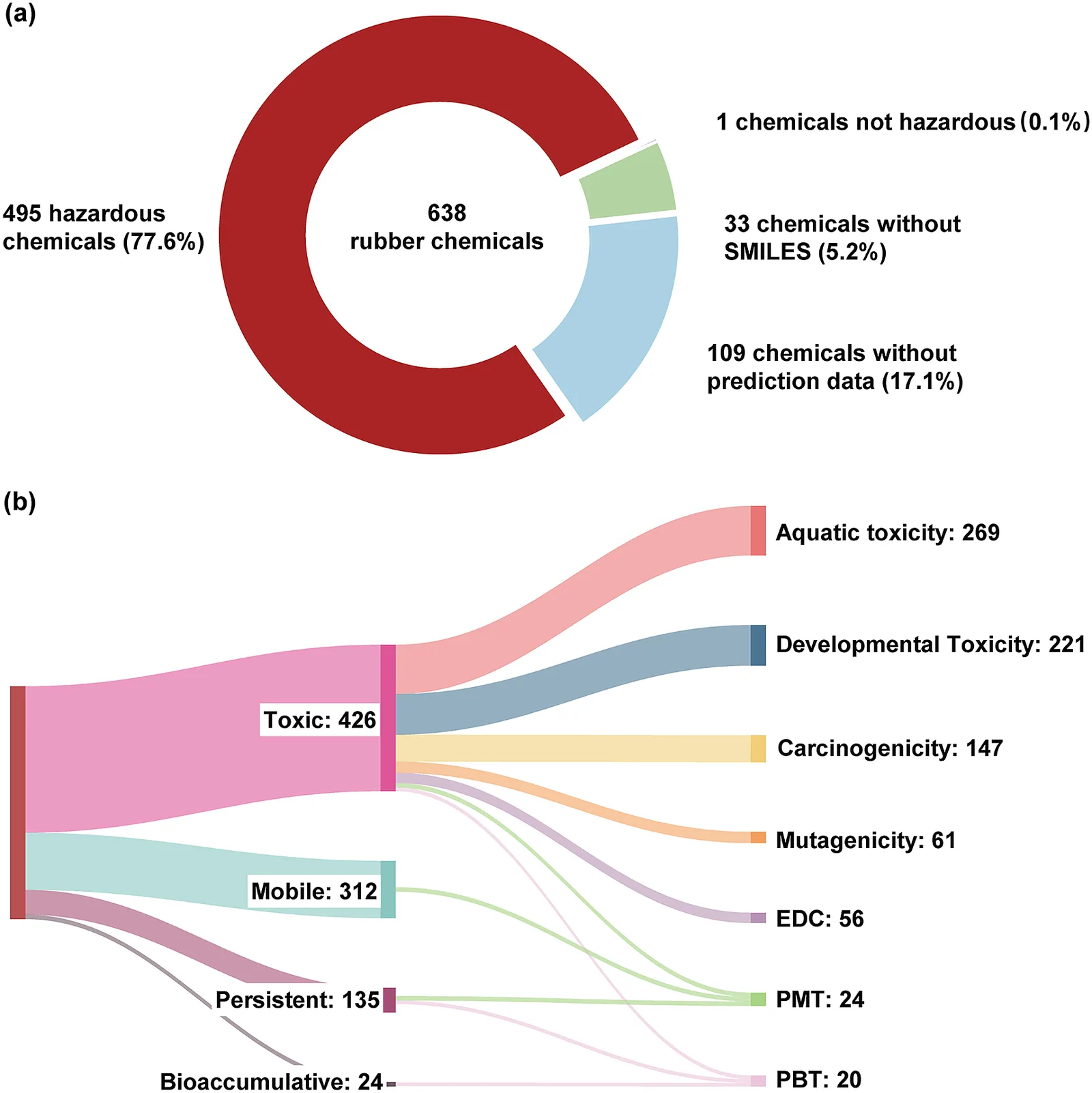
Data availability screening and composition of P, B, M, and T hazard signals for rubber-related chemicals based on the mean-model approach. (a) Outcomes for 638 chemicals, including chemicals with unavailable structural information, chemicals with unavailable prediction data, and chemicals that could be evaluated. (b) Composition of P, B, M, and T signals and their sub-endpoints among evaluable chemicals, together with the numbers of PBT substances and PMT substances identified based on the corresponding criteria combinations. SMILES, simplified molecular input line entry system; PBT, persistent, bioaccumulative, and toxic; PMT, persistent, mobile, and toxic; EDC, endocrine-disrupting chemical.

**Fig. 3:**
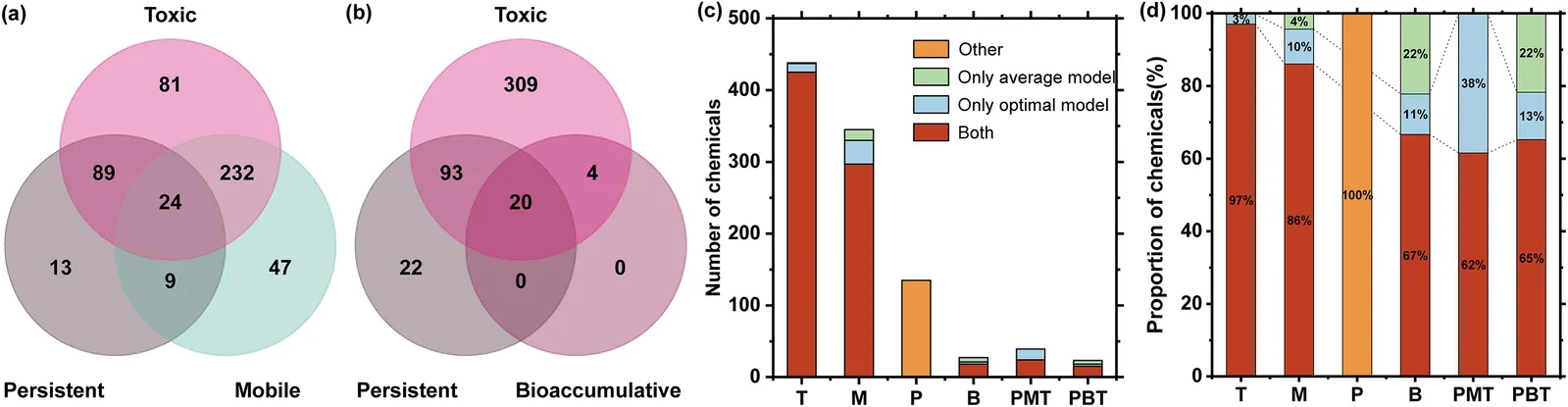
Consistency between the mean-model and selected-model in identifying PMT and PBT substances. (a) PMT and (b) PBT substances identified by the mean-model. (c) Numbers of chemicals classified in each hazard dimension and PMT/PBT groups by both, average-only, or optimal-model approaches. (d) Corresponding proportions for each category.

Based on the endpoint classification criteria described in Section 2.2 and the two endpoint integration strategies in Section 2.3, we performed PBT and PMT identification under both the mean-model and selected-model frameworks. Using the mean-model approach as an example, Fig. 2 summarizes the screening results for 638 rubber-related chemicals. After excluding 33 chemicals lacking reliable structural information (5.2%) and 109 chemicals without useable prediction data (17.1%), 496 chemicals remained evaluable. Among them, 495 chemicals met at least one of the P, B, M, or T criteria (Fig. 2a). Toxicity and mobility signals were most common. Specifically, 426 chemicals were classified as T and 312 as M, whereas persistence and bioaccumulation were less frequent, with 135 and 24 chemicals, respectively (Fig. 2b). This pattern was mainly driven by the broad endpoint coverage of T and the conservative nature of current *K*_oc_-based mobility screening. Therefore, a trigger for at least one of P, B, M, or T should be interpreted as a conservative screening signal rather than uniformly great concern across chemicals. Under the mean-model approach, 24 PMT substances and 20 PBT substances were identified (Fig. 3a and b). Under the selected-model approach, 39 PMT substances and 18 PBT substances were identified. Taking the union of both approaches yielded 23 PBT and 39 PMT candidate substances.

Across the chemicals discussed above that ranked within the top 50 by ToxPi under either approach, a total of 60 chemicals were identified. We further included 22 additional substances that triggered prioritization by meeting the criteria for PBT substances or PMT substances. This expanded the candidate list and provided a more comprehensive set of priority chemicals. The overlap between the two approaches was compared at the endpoint level and at the level of the PBT and PMT sets (Fig. 3c and d). The shared identification proportions for the toxicity and mobility endpoints were 97% and 86%, respectively, indicating high consistency between approaches. In contrast, the shared identification proportion for the bioaccumulation endpoint decreased to 67%, suggesting greater sensitivity to differences in model applicability domains and training data. The PMT and PBT sets also showed reasonable robustness. The proportions simultaneously identified by both approaches were approximately 62% for PMT and 65% for PBT, and the remaining substances were contributed by a single approach. Comparison with external lists showed that 11 of the 23 PBT substances had already been included in U.S. EPA and/or REACH-related PBT lists, and four PMT substances were consistent with the priority PMT list of the German Environment Agency (UBA). Overall, the two strategies were complementary for PBT and PMT identification. The union-based candidate list maintained a relatively high level of agreement while reducing the risk of omissions associated with reliance on a single approach.

### 3.4 Integrated hazard-exposure prioritization of 12 specific rubber additives

**Fig. 4:**
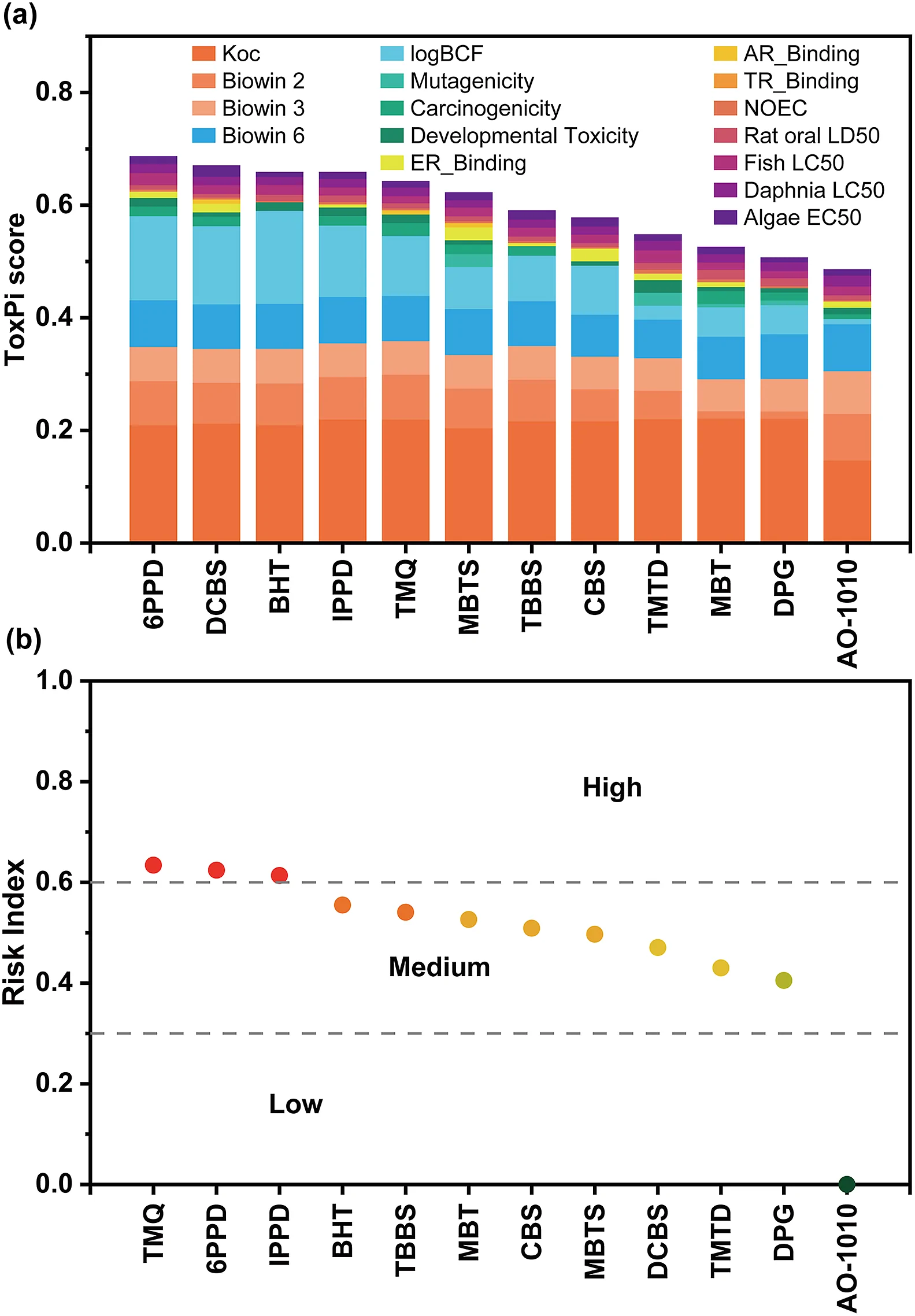
Hazard-based prioritization and exposure-informed RI re-ranking of the 12 specific rubber additives. (a) Hazard ranking based on ToxPi scores, together with the corresponding endpoint contributions. (b) Exposure-informed RI ranking after incorporating formulation loading and migration potential as exposure proxies. ToxPi, Toxicological Priority Index; RI, Risk Index; AR, androgen receptor; TR, thyroid receptor; ER, estrogen receptor; NOEC, no observed effect concentration.

Table 2 summarizes the functional categories of typical additives used in the rubber industry and their commonly reported formulation ranges. Based on this information, representative chemicals were selected for integrated risk prioritization. First, under the ToxPi scoring (Fig. 4a), N-(1,3-dimethylbutyl)-N′-phenyl-p-phenylenediamine (6PPD) showed the highest composite hazard score, followed by N,N-dicyclohexyl-2-benzothiazolesulfenamide (DCBS) and 2,6-di-tert-butyl-4-methylphenol (BHT). N-isopropyl-N′-phenyl-1,4-phenylenediamine (IPPD) and 2,2,4-trimethyl-1,2-dihydroquinoline (TMQ) formed a second tier. The remaining chemicals ranked lower in the following order: 2,2′-dibenzothiazyl disulfide (MBTS), N-tert-butyl-2-benzothiazolesulfenamide (TBBS), N-cyclohexyl-2-benzothiazolesulfenamide (CBS), tetramethylthiuram disulfide (TMTD), 2-mercaptobenzothiazole (MBT), and diphenylguanidine (DPG), whereas pentaerythritol tetrakis[3-(3,5-di-tert-butyl-4-hydroxyphenyl)propionate] (AO-1010) had the lowest score. Overall, amine antioxidants, including 6PPD, IPPD, and TMQ, contributed relatively more to the hazard component. Among accelerators, DCBS and MBTS ranked higher due to higher ToxPi scores, followed by TBBS and CBS, whereas TMTD, MBT, and DPG showed comparatively lower hazard contributions. The hindered phenolic antioxidant AO-1010 ranked last in the hazard-based assessment, indicating a relatively small contribution to the composite hazard profile under the endpoint set considered in this study.

**Table 2:** Representative formulation loading ranges of additives from typical functional categories in rubber formulations.

Functional category	CAS	Abbreviation	Formulation range (phr)
Accelerators	149-30-4	MBT	0.6–2.0
	137-26-8	TMTD	0.0–1.0
	95-31-8	TBBS	1.0–2.0
	120-78-5	MBTS	1.0–2.0
	95-33-0	CBS	1.0–2.0
	4979-32-2	DCBS	0.5–1.0
	102-06-7	DPG	0.3–0.5
Antioxidants	26780-96-1	TMQ	0.5–2.0
	793-24-8	6PPD	1.0–3.0
	128-37-0	BHT	0.3–1.0
	101-72-4	IPPD	1.0–2.0
	6683-19-8	AO-1010	0.3–1.0
Crosslinking agents	80-43-3	DCP	3.0–5.0
	101-37-1	TAC	1.0–2.0
Vulcanization activators	57-11-4	Stearic acid	0.5–2.0
Fillers and reinforcing agents	40372-72-3	TESPT	0.5–3.0

After accounting for typical formulation loading and migration potential (Fig. 4b), the integrated risk ranking was reshuffled. TMQ moved from a mid to high position in the hazard-based ranking to the highest priority in the integrated risk assessment. Its typical formulation loading range is 0.5 to 2.0 phr, with a midpoint value of 1.25 phr, indicating both relatively high loading and widespread use. Recent non-target screening studies on rubber aging chemistry have reported abundant TMQ-related transformation signals during the aging of tire and artificial turf rubber particles. This observation suggests that TMQ may generate a substantial and structurally diverse suite of transformation products during environmental weathering [64]. In addition, studies combining quantum chemical calculations with receptor-binding simulations have indicated that TMQ-derived radicals and their reaction products exhibit the highest potential interference with thyroid hormone receptors in aquatic organisms among several commonly used tire antioxidant derivatives. The predicted interference tendency also increased with trophic level, which mechanistically supports the need for more systematic toxicity identification and prioritized monitoring of TMQ and its transformation products [65].

6PPD ranked highest in the hazard component and remained second after incorporating exposure, reflecting the combined effect of high hazard and high exposure potential. This antioxidant is frequently used at relatively high loadings in tire tread compounds, with typical formulations reaching 2–3 phr, and it is currently one of the most widely scrutinized chemicals in rubber products. Multiple studies have reported toxic effects of 6PPD on aquatic organisms. For example, 6PPD can markedly impair zebrafish embryonic development and reduce larval locomotor activity [66]. It may also induce hepatotoxicity and metabolic disturbances in mammals, suggesting potential implications for human health [67]. During tire wear, rubber particles can be transformed in the environment to 6PPD quinone, and stormwater runoff can transport this transformation product into receiving waters, causing acute mortality in fish such as coho salmon [68]. Notably, coho salmon captured during heavy rainfall events can die within only a few hours [10]. In the 27 June 2025 amendment to the Restrictions Roadmap, PPD was included in Annex I, and entry 6.2 specifically covers 6PPD and related substances. The amendment also indicates that restrictions are under consideration for 6PPD and related compounds in rubber tires and other rubber products, reflecting growing regulatory scrutiny of this substance group [69]. Therefore, in scenarios dominated by tire wear and road runoff, the integrated risk priority of 6PPD may warrant further upward adjustment. IPPD ranked among the highest in the hazard component and remained within the high-risk range after exposure was incorporated. Its typical formulation range is 1.0–2.0 phr, with a midpoint value of 1.5 phr, indicating that its risk contribution is also jointly driven by intrinsic hazard and formulation loading.

Industry statistics further show that 6PPD and TMQ are the two highest-production antidegradants in China. In 2024, their outputs were 2.242 × 10^5^ t and 1.449 × 10^5^ t, respectively [70]. Together, they accounted for approximately 81.7% of the total output of antidegradants of 4.516 × 10^5^ t in that year [7]. This pattern implies broader coverage in practical applications and a larger total mass entering rubber matrices, which is consistent with their high integrated risk priorities observed in this study.

In contrast, AO-1010 was consistently classified as a low-hazard chemical under both ranking approaches. Its integrated risk remained low because it is typically used at relatively low loadings and exhibits high hydrophobicity (log *K*_oc_ > 6) and a relatively large molecular weight, which together imply low volatility and reduced mobility. These properties are expected to limit environmental release. AO-1010 is commonly used in light-colored or non-staining formulations, such as cable sheaths and medical rubber, and it has been included in the Safer Chemical Ingredients List (SCIL) as meeting safer selection criteria for its functional ingredient class. Accordingly, AO-1010 fell within the low-risk region (Fig. 4b), which is consistent with its current regulatory status.

To extend the interpretation beyond the 12 representative additives included in the RI analysis, we further screened the 82 high-priority chemicals and selected a subset of intentional additives that are compatible with the present concentration-migration framework but currently lack publicly accessible formulation-level data (Table S7). These substances should be prioritized for future concentration characterization. By contrast, crosslinking agents, reinforcing fillers, and other reactive/particulate components were not retained as candidates for this framework because their occurrence and release are more strongly governed by reaction processes, matrix incorporation, particulate forms, or abrasion-related pathways than by small-molecule migration.

Overall, introducing the risk index enabled the prioritization to jointly reflect intrinsic hazard and the potential exposure level under realistic use scenarios, thereby reducing the bias of hazard-only ranking and improving the practical relevance of the results. In products characterized by high loadings and high exposure potential, such as tires and shoe soles, the priority of substances such as TMQ and 6PPD increased further. In contrast, substances such as AO-1010, which have low typical formulation loading and low migration-driven release potential, remained associated with low integrated risk. Together with the candidate list provided in Table S7, these results provide a more targeted basis for future concentration characterization, substitution assessment, and regulatory decision-making in specific rubber product scenarios.

### 3.5 Study limitations

Nevertheless, several limitations of the present framework should be acknowledged. First, due to the applicability-domain limitations of QSAR models, the current framework cannot provide stable and reliable evaluations for substances with uncertain structures or variable composition, such as UVCBs, polymers, most inorganic substances, organometallic compounds, and chemicals lacking useable prediction data. Accordingly, the present screening outcomes are primarily representative of structurally defined chemicals amenable to QSAR-based evaluation, rather than the full spectrum of rubber-related chemicals. Expanding future software tools and assessment methods to better cover complex substances remains an important direction for improving the representativeness of the framework.

Second, because of constraints in the availability of public databases and regulatory lists, the number of rubber-related chemicals and the completeness of functional labels included in this study may not fully represent real-world supply chains. In addition, chemicals shared across different material categories may lead to functional misclassification. Future work should combine public data integration with real industry data for cross-checking and completion, incorporate material-specific scenarios, and use information to improve chemical coverage and labeling accuracy.

Third, the present framework should be regarded as a screening-level relative prioritization tool rather than a definitive concentration- or dose-based quantitative risk assessment [71]. Future work should therefore focus on high-priority additives through targeted quantification in rubber products and recycled rubber materials [72], time-resolved leaching or release experiments to derive actual release kinetics [73], and comparison with monitoring data in relevant environmental matrices [9]. In parallel, the predicted hazards should be cross-checked against available in vitro and in vivo toxicity evidence. For chemicals with suitable dose-response data, benchmark dose modeling could further provide more robust toxicity thresholds. Ultimately, integrating empirically constrained release data, predicted environmental concentrations or human exposure doses, and toxicity thresholds for estimating risk quotients or margins of safety would provide a logical next step from screening-level prioritization toward quantitative risk management of rubber additives [74,75].

Finally, the current framework focuses on intentionally added parent additives and does not systematically account for transformation products (TPs) and reaction by-products generated during use, aging, and environmental weathering. This oversight is of high environmental relevance, as TPs may exhibit higher toxicity, mobility, or persistence than their parent compounds. Future work should build on the current prioritization of high-priority parent additives and integrate transformation-pathway prediction tools (e.g., BioTransformer, CTS, or enviPath) with in silico toxicity profiling to identify environmentally plausible TPs [76]. Ultimately, coupling these computational predictions with suspect or non-target screening and subsequent experimental confirmation will be essential to comprehensively capture the long-term risks of rubber products.

## 4 Conclusions

This study developed an integrated chemical screening framework for rubber applications that links data preparation with prioritization decisions. Using an automated workflow, we systematically curated and harmonized multiple authoritative databases to establish a rubber additive dataset with structural identifiers and function labels. On this basis, multi-model and multi-endpoint QSAR prediction was carried out, with uncertainty control applied during endpoint assignment. Two parallel strategies, the mean-model approach and the selected-model approach, were applied to assess persistence, bioaccumulation, mobility, and toxicity. We first established a robust hazard-based prioritization and further complemented this ranking by identifying additional chemicals of concern using PBT and PMT criteria. This process yielded a hazard-based priority candidate list comprising 82 chemicals. For 12 specific rubber additives with publicly accessible formulation ranges, representative formulation loading and a molecular-weight-based relative migration potential metric were further incorporated to construct an exposure factor and the integrated RI. The two endpoint integration strategies showed strong agreement in overall ranking while remaining complementary for a small set of endpoint-sensitive chemicals. After exposure was included, chemicals with higher typical loadings but moderate hazards, such as TMQ, increased in priority. In contrast, chemicals with lower use levels or weaker migration potential, such as AO-1010, decreased in priority. Chemicals such as 6PPD, characterized by both high hazard and moderate to high formulation, remained near the top. These results make the prioritization more aligned with practical industrial conditions and provide an actionable evidence base to support market access decisions, substitution assessment, and regulatory early warning for rubber additives.

## CRediT authorship contribution statement

**Sihan Wang:** Writing – review & editing, Writing – original draft, Visualization, Software, Methodology, Investigation, Formal analysis, Data curation, Conceptualization. **Wanting Dai:** Validation, Formal analysis, Data curation. **Bin Wang:** Writing – review & editing, Writing – original draft, Supervision, Methodology, Funding acquisition, Conceptualization. **Liping Heng:** Writing – review & editing, Writing – original draft, Supervision.

## Declaration of competing interest

The authors declare that they have no known competing financial interests or personal relationships that could have appeared to influence the work reported in this paper.
