# Supplementary material for: Integrated risk prioritization of rubber additives based on hazard ranking and exposure characterization
Source: Eco Environ Health. 2026 Sep 2;5(4):100282. doi: 10.1016/j.eehl.2026.100282 (PMC13597201; doi:10.1016/j.eehl.2026.100282)
Supplement: Supplementary file 1 [file mmc1.docx]

Supporting information

Fig. S1. Python code for constructing the rubber additive database from multiple data sources.

Table S1. Detailed information on the computational models used to predict each endpoint.

| **Criteria** | | **Attributes** | **Weight** | **Software** | ***In silico* models** |
| --- | --- | --- | --- | --- | --- |
| **Persistence** | | Biowin2 | 1/12 | EPI SUITE | BIOWIN |
|  |  | Biowin3 | 1/12 |  | BIOWIN |
|  |  | Biowin6 | 1/12 |  | BIOWIN |
| **Bioaccumulation** | | Log BCF | 1/4 | EPI SUITE | BCFBAF model |
|  |  |  |  | VEGA | BCF model (CAESAR) |
|  |  |  |  |  | BCF model (Meylan) |
|  |  |  |  |  | BCF model (Arnot-Gobas) |
|  |  |  |  |  | BCF model (KNN-Read-Across) |
| **Mobility** | | Log Koc | 1/4 | EPI SUITE | KOCWIN MCI Method |
|  |  |  |  |  | KOCWIN KOW Method |
|  |  |  |  | OPERA | KOC model (OPERA)-prediction |
| **Toxicity** | **Ecotoxicological effects** | Fish Acute LC50 | 1/44 | VEGA | Fish Acute (LC50) Toxicity model (IRFMN) |
|  |  |  |  |  | Fish Acute (LC50) Toxicity model (NIC) |
|  |  |  |  |  | Fish Acute (LC50) Toxicity model (KNN-Read-Across) |
|  |  |  |  |  | Fish Acute (LC50) Toxicity model (IRFMN-Combase) |
|  |  |  |  |  | Fathead Minnow LC50 96h (EPA) |
|  |  |  |  |  | Fathead Minnow LC50 model (KNN-IRFMN) |
|  |  |  |  | TEST | Fathead Minnow LC50 96h_Consensus |
|  |  | Daphnia Magna LC50 | 1/44 | VEGA | Daphnia Magna LC50 48h (EPA) |
|  |  |  |  |  | Daphnia Magna LC50 48h (DEMETRA) |
|  |  |  |  | TEST | Daphnia Magna LC50 48h_Consensus |
|  |  | Algae Acute EC50 | 1/44 | VEGA | Algae Acute (EC50) Toxicity model (IRFMN) |
|  |  |  |  |  | Algae Acute (EC50) Toxicity model (ProtoQSAR-Combase) |
|  | **Human health effects** | Repeated dose toxicity | 1/44 | VEGA | NOAEL_VEGA_IRFMN-CORAL |
|  |  | Mutagenicity | 1/44 | VEGA | Mutagenicity (Ames test) model (CAESAR) |
|  |  |  |  |  | Mutagenicity (Ames test) model (ISS) |
|  |  |  |  |  | Mutagenicity (Ames test) model (SarPy-IRFMN) |
|  |  |  |  |  | Mutagenicity (Ames test) model (KNN-Read-Across) |
|  |  | Carcinogenicity | 1/44 | VEGA | Carcinogenicity model (CAESAR) |
|  |  |  |  |  | Carcinogenicity model (ISS) |
|  |  |  |  |  | Carcinogenicity model (IRFMN-ISSCAN-CGX) |
|  |  |  |  |  | Carcinogenicity model (IRFMN-Antares) |
|  |  | Developmental Toxicity | 1/44 | VEGA | Developmental Toxicity model (CAESAR) |
|  |  |  |  |  | Developmental/Reproductive Toxicity library (PG) |
|  |  |  |  | TEST | Pred_Consensus |
|  |  | Estrogen binding | 1/44 | VEGA | Estrogen Receptor-mediated effect (IRFMN-CERAPP) |
|  |  |  |  |  | Estrogen Receptor Relative Binding Affinity model (IRFMN) |
|  |  |  |  | OCHEM | Est_OCHEM_PublicID300 |
|  |  |  |  |  | Est_OCHEM_PublicID518 |
|  |  | Androgen binding | 1/44 | VEGA | Androgen Receptor-mediated effect (IRFMN-COMPARA) |
|  |  |  |  | OCHEM | And_OCHEM_PublicID301 |
|  |  |  |  |  | And_OCHEM_PublicID516 |
|  |  | Thyroid binding | 1/44 | OCHEM | Thy_OCHEM_PublicID302 |
|  |  | Rat oral LD 50 | 1/44 | TEST | Pred_Nearest neighbor |
|  |  |  |  |  | Pred_Hierarchical clustering |

S1. Experimental data support and endpoint-specific model evaluation for the selected-model approach

S1.1 Experimental data sources and harmonization

Experimental data used for endpoint-specific model comparison were compiled from software-associated experimental databases. For endpoints with sufficient experimental support, the numbers of experimental records included in model comparison were 65 for log Koc, 121 for log BCF, 179 for mutagenicity, 74 for carcinogenicity, 141 for estrogen binding, 193 for Rat oral LD50, 113 for Fish Acute LC50, and 81 for Daphnia magna LC50. For a given endpoint, experimental values provided by different software sources were generally consistent. In the few cases where discrepancies were observed, the differences were usually small, and the mean value was used as the reference for model comparison. This data integration procedure minimized the possibility that endpoint-specific model selection would be driven by experimental values originating predominantly from a single software source.

Fig. S2. Predictive performance of QSAR models for log BCF other than the best-performing model: (a) CAESAR, (b) Meylan, (c) BCFBAF, and (d) Arnot-Gobas.

Fig. S3. Predictive performance of QSAR models for log Koc other than the best-performing model: (a) KOCMIN (KOW method) and (b) KOCWIN (MCI method).

Fig. S4. Predictive performance of QSAR models for Daphnia magna LC50 other than the best-performing model: (a) Daphnia magna LC50 (48 h, DEMETRA) assessment and (b) TEST consensus.

Fig. S5. Predictive performance of QSAR models for rat oral LD50 other than the best-performing model: (a) Pred_Nearest neighbor and (b) TEST consensus.

Fig. S6. Predictive performance of QSAR models for fish acute LC50 other than the best-performing model: (a) IRFMN assessment, (b) IRFMN-Combase assessment, (c) KNN-Read-Across assessment, (d) KNN-IRFMN assessment, (e) TEST consensus, and (f) NIC assessment.

S1.2 Evaluation of continuous endpoints

For continuous endpoints, including log Koc, log BCF, Rat oral LD50, Fish Acute LC50, and Daphnia magna LC50, predicted values were fitted to experimental values using linear regression. Model performance was quantified using the coefficient of determination (R^2^), which was used to measure goodness of fit between predicted and experimental values. A higher R^2^ value indicates better agreement and stronger predictive performance.

S1.3 Evaluation of categorical endpoints

To evaluate the performance of different QSAR classification models in predicting qualitative endpoints, including carcinogenicity, mutagenicity, and estrogen receptor binding activity, three key confusion-matrix-based metrics were used: recall, Matthews correlation coefficient (MCC), and balanced accuracy (BA). These metrics quantify the accuracy and robustness of models in identifying positive and negative samples, thereby supporting the selection of the optimal model for each endpoint.

The confusion matrix includes four core parameters:

1. True positive (TP): predicted positive and actually positive
2. False positive (FP): predicted positive but actually negative
3. True negative (TN): predicted negative and actually negative
4. False negative (FN): predicted negative but actually positive

Based on these terms, three statistical metrics were calculated for model evaluation:

(1) Recall

Recall reflects the model’s ability to identify positive (toxic) samples and is sensitive to false negatives. It is calculated as:

(2) MCC

MCC jointly considers TP, TN, FP, and FN, and is robust under class-imbalanced conditions. Its value ranges from −1 to 1. The formula is:

An MCC value closer to 1 indicates more stable and reliable model predictions.

(3) BA

Because several qualitative endpoints in this study exhibit substantial class imbalance in the reference data, for example, for estrogen receptor binding activity where positive samples are far fewer than negative samples, conventional accuracy cannot fully capture model performance and may lead to majority-class bias. Therefore, balanced accuracy was used as a more discriminative metric. BA accounts for performance on both positive and negative classes and provides a more comprehensive measure of classification performance under imbalanced conditions. It is calculated as:

For each endpoint, the best-performing model was selected based on the combined performance across the three metrics. Priority was given to models with high recall and balanced accuracy and with consistently strong MCC values, to ensure high sensitivity for toxic-positive compounds and overall model stability.

**Fig. S7. Performance evaluation of three qualitative models for the carcinogenicity endpoint other than the best-performing model.** Confusion matrices for (a) CAESAR assessment, (b) ISS assessment, and (c) IRFMN-Antares assessment. (d) Comparison of Recall, MCC, and balanced accuracy across the four models, showing that the ISSCAN-CGX assessment achieved the best overall performance.

**Fig. S8. Performance evaluation of three qualitative models for the estrogen receptor binding activity endpoint other than the best-performing model.** Confusion matrices for (a) Estrogen Receptor Relative Binding Affinity model (IRFMN), (b) Est_OCHEM_PublicID300, and (c) Est_OCHEM_PublicID518. (d) Comparison of Recall, MCC, and balanced accuracy across the four models, showing that the Estrogen Receptor-mediated effect (IRFMN-CERAPP) achieved the best overall performance.

**Fig. S9. Performance evaluation of three qualitative models for the mutagenicity endpoint other than the best-performing model.** Confusion matrices for (a) CAESAR assessment, (b) ISS assessment, and (c) SarPy-IRFMN assessment. (d) Comparison of Recall, MCC, and balanced accuracy across the four models, showing that the Mutagenicity (Ames test) model (KNN-Read-Across) achieved the best overall performance.

S2. Stability analysis of the top 10% high-risk chemicals under alternative ranking scenarios

To examine the robustness of the high-risk ranking results, four ranking scenarios were compared, namely the Mean-model approach (MEAN), the Selected-model approach (SEL), the Best-case approach (BEST), and the Worst-case approach (WORST). MEAN and SEL correspond to the two hazard integration strategies used in the main analysis. BEST and WORST were constructed to represent the lower and upper bounds of model-driven uncertainty. For each chemical and each endpoint, BEST used the least hazardous prediction among the available models, whereas WORST used the most hazardous prediction. All approaches were processed using the same normalization and ranking workflow as that used in the main analysis.

Ranking stability was evaluated using three complementary metrics. First, the Spearman rank correlation coefficient (𝜌) was used to assess the overall agreement of ranking orders across all chemicals. For two ranking lists, the coefficient was calculated as:

where *d_i_* is the rank difference of chemical 𝑖 between two approaches and 𝑛 is the total number of chemicals. A value of 𝜌 closer to 1 indicates stronger agreement between the two rankings. Second, the top 10% overlap was used to evaluate the consistency of the high-risk head group. For two approached with top 50 chemical sets 𝑇*_a_* and 𝑇*_b_*, the overlap was defined as:

Third, the top 10% retention in the top 15% tier was used to evaluate whether chemicals identified as high risk under one approach remained within the broader high-risk tier under alternative approaches. For a reference top 50 set *T*_10, re_*_f_* and an alternative top 75 set *T*_15, re_*_f_*, the retention was defined as:

To characterize chemicals near the top 10% cut-off, borderline chemicals were defined as those ranked from 40 to 60 in the reference scenario. Their stability was evaluated by examining whether they remained within the top 15% and top 20% tiers under the alternative approaches.

Table S2. Rankings of chemicals included in the top 10% under the Mean-model approach or the Selected-model approach across the four ranking approaches.

| **CAS No** | **Rank** | | | |
| --- | --- | --- | --- | --- |
|  | **AVG** | **SEL** | **BEST** | **WORST** |
| 11097-69-1 | 1 | 1 | 5 | 1 |
| 53469-21-9 | 2 | 2 | 4 | 3 |
| 118-74-1 | 3 | 6 | 2 | 12 |
| 1163-19-5 | 4 | 9 | 3 | 6 |
| 2385-85-5 | 5 | 4 | 1 | 23 |
| 133-49-3 | 6 | 3 | 8 | 13 |
| 59447-57-3 | 7 | 7 | 34 | 2 |
| 207-08-9 | 8 | 17 | 10 | 20 |
| 205-82-3 | 9 | 15 | 12 | 17 |
| 193-39-5 | 10 | 14 | 27 | 7 |
| 205-99-2 | 11 | 16 | 11 | 24 |
| 108171-26-2 | 12 | 57 | 37 | 8 |
| 608-93-5 | 13 | 12 | 7 | 45 |
| 133-14-2 | 14 | 8 | 21 | 14 |
| 732-26-3 | 15 | 13 | 6 | 46 |
| 3846-71-7 | 16 | 11 | 17 | 28 |
| 93-46-9 | 17 | 29 | 48 | 4 |
| 3194-55-6 | 18 | 19 | 13 | 26 |
| 134237-52-8 | 19 | 20 | 14 | 27 |
| 85535-84-8 | 20 | 64 | 56 | 19 |
| 101-14-4 | 21 | 26 | 9 | 47 |
| 27417-40-9 | 22 | 25 | 45 | 18 |
| 192-97-2 | 23 | 36 | 30 | 32 |
| 3864-99-1 | 24 | 21 | 22 | 44 |
| 79-94-7 | 25 | 24 | 15 | 62 |
| 556-67-2 | 26 | 23 | 18 | 52 |
| 3081-14-9 | 27 | 50 | 55 | 9 |
| 74-31-7 | 28 | 31 | 20 | 69 |
| 50-32-8 | 29 | 28 | 16 | 86 |
| 101-87-1 | 30 | 59 | 44 | 38 |
| 26545-49-3 | 31 | 119 | 78 | 10 |
| 25973-55-1 | 32 | 34 | 79 | 30 |
| 91-94-1 | 33 | 33 | 23 | 65 |
| 632-79-1 | 34 | 18 | 80 | 21 |
| 4130-42-1 | 35 | 30 | 24 | 80 |
| 56-55-3 | 36 | 35 | 19 | 87 |
| 198-55-0 | 37 | 38 | 28 | 68 |
| 140-66-9 | 38 | 32 | 25 | 74 |
| 6267-02-3 | 39 | 55 | 47 | 39 |
| 793-24-8 | 40 | 58 | 38 | 51 |
| 1889-67-4 | 41 | 56 | 46 | 40 |
| 64359-81-5 | 42 | 63 | 52 | 29 |
| 58-36-6 | 43 | 27 | 106 | 25 |
| 129-00-0 | 44 | 43 | 26 | 103 |
| 218-01-9 | 45 | 42 | 29 | 90 |
| 27193-28-8 | 46 | 54 | 39 | 55 |
| 3380-34-5 | 47 | 41 | 31 | 71 |
| 32588-76-4 | 48 | 60 | 67 | 22 |
| 3147-75-9 | 49 | 48 | 71 | 41 |
| 56-35-9 | 50 | 80 | 42 | 88 |
| 36437-37-3 | 51 | 37 | 92 | 36 |
| 119-93-7 | 53 | 49 | 33 | 70 |
| 21850-44-2 | 56 | 5 | 118 | 5 |
| 25637-99-4 | 57 | 40 | 97 | 15 |
| 88-27-7 | 61 | 22 | 63 | 33 |
| 101-72-4 | 63 | 39 | 65 | 58 |
| 90-30-2 | 64 | 46 | 43 | 85 |
| 10081-67-1 | 71 | 44 | 125 | 43 |
| 97416-84-7 | 77 | 10 | 149 | 11 |
| 31851-03-3 | 157 | 47 | 271 | 73 |

Table S3. Pairwise stability metrics among the four ranking scenarios for the top 10% high-risk chemicals.

| **APPROACH** | **MEAN** | **SEL** | **BEST** | **WORST** |
| --- | --- | --- | --- | --- |
| **MEAN** | — | 0.953 | 0.957 | 0.957 |
| **SEL** | 39/50 (78.0%) | — | 0.911 | 0.924 |
| **BEST** | 41/50 (82.0%) | 36/50 (72.0%) | — | 0.846 |
| **WORST** | 35/50 (70.0%) | 33/50 (66.0%) | 26/50 (52.0%) | — |

Note: Values in the upper triangle are Spearman rank correlation coefficients, indicating overall rank agreement across all 496 chemicals. Values in the lower triangle are overlaps of the top 10% chemicals, reported as n/50 (%), indicating the number and percentage of chemicals shared by the top 50 positions in each pair of approaches.

Table S4 shows that the overall ranking remained highly consistent across the four approaches. with 𝜌 values ranging from 0.846 to 0.957. The agreement between MEAN and SEL was particularly high, with 𝜌 = 0.953. Comparisons of MEAN or SEL with BEST or WORST also remained high, with 𝜌 values ranging from 0.911 to 0.957. The lowest agreement was observed between BEST and WORST, with 𝜌 = 0.846, which represents the maximum contrast under the two extreme perturbation settings.

At the level of the high-risk head group, the top 10% overlap between MEAN and SEL was 39/50, corresponding to 78.0%. The overlap between MEAN and BEST was 41/50, and that between MEAN and WORST was 35/50. The corresponding overlaps for SEL were 36/50 with BEST and 33/50 with WORST. These results indicate that the top-ranked chemicals remained broadly consistent under alternative approaches. Although the overlap between BEST and WORST was lower at 26/50, this comparison represents the maximum contrast obtained by simultaneously assigning the least hazardous and most hazardous predictions across all endpoints.

Further evaluation of tier retention showed that the high-risk head group remained stable under alternative approaches. Using MEAN as the reference, 48/50 chemicals remained within the top 15% tier under SEL, 46/50 under BEST, and 44/50 under WORST, corresponding to 96.0%, 92.0%, and 88.0%, respectively. Using SEL as the reference, the corresponding retention rates were 94.0%, 80.0%, and 86.0% under MEAN, BEST, and WORST, respectively. These results indicate that most chemicals identified in the top 10% under the routine MEAN and SEL approaches remained within the broader high-risk tier even under extreme perturbation settings.

Chemicals near the top 10% threshold showed greater local rank sensitivity, but they largely remained within the upper part of the ranking. For borderline chemicals defined from ranks 40 to 60 under MEAN, the proportions retained within the top 15% under SEL, BEST, and WORST were 90.5%, 66.7%, and 76.2%, respectively. When the broader top 20% tier was considered, these proportions increased to 100.0%, 81.0%, and 90.5%. For borderline chemicals defined from ranks 40 to 60 under SEL, the proportions retained within the top 15% under MEAN, BEST, and WORST were 85.7%, 76.2%, and 76.2%, respectively, and the corresponding proportions within the top 20% were 85.7%, 81.0%, and 90.5%. Therefore, scenario perturbation mainly affected the local ordering of chemicals around the top 10% cut-off, while most of these chemicals still remained within the high-risk front section of the ranking.

Table S4. Typical additive categories and representative formulation loading ranges for different rubber product types (e.g., tires, shoe soles, hoses, and vibration-damping components).

| **Additive** | **Tires (phr)** | **Vibration-damping rubber (phr)** | **Shoe soles (phr)** | **Hoses (phr)** | **Overall (phr)** |
| --- | --- | --- | --- | --- | --- |
| 6PPD | 1.0–3.0 | 1.5–2.5 | 0 | 0 | 1.0–3.0 |
| TMQ | 0.5–1.5 | 1.5–2.0 | 0.3–1.5 | 1.0–2.0 | 0.5–2.0 |
| TBBS(NS) | 1.0–1.75 | 1.5–2.0 | 1.5–2.0 | n | 1.0–2.0 |
| DPG(D) | 0.3–0.5 | n | 0.3 | n | 0.3–0.5 |
| TMTD(TT) | 0.1–0.3 | n | n | 0–1.0 | 0–1.0 |
| TESPT | 0.5–3.0 | n | 2.0–3.0 | n | 0.5–3.0 |
| DCBS(DZ) | 0.5–1.0 | n | n | n | 0.5–1.0 |
| CBS(CZ) | 1.0–1.5 | 1.5–2.0 | n | n | 1.0–2.0 |
| MBTS(DM) | n | n | 1.0–2.0 | 1.0–1.5 | 1.0–2.0 |
| IPPD | 1.0–2.0 | 1.5–2.0 | 0 | 0 | 1.0–2.0 |
| MBT(M) | n | n | n | 0.6–2.0 | 0.6–2.0 |
| DCP | n | n | n | 3.0–5.0 | 3.0–5.0 |
| TAC | n | n | n | 1.0–2.0 | 1.0–2.0 |
| Stearic acid | 0.5–2.0 | 1.0–1.5 | 0.5–1.5 | 0–1.0 | 0.5–2.0 |
| AO-1010 | n | n | 0.3–1.0 | n | 0.3–1.0 |
| BHT | n | n | 0.3–1.0 | n | 0.3–1.0 |

Note: n indicates not used or not reported

Table S5. Sensitivity comparison of Risk Index values and rankings under multiplicative coupling and additive weighted-sum coupling

| **Chemical** | **RI_mul** | **RI_aws** | **ΔRank** |
| --- | --- | --- | --- |
| TMQ | 0.6341 | 0.8146 | 0 |
| 6PPD | 0.6242 | 0.7979 | 0 |
| IPPD | 0.6135 | 0.7950 | 0 |
| BHT | 0.5550 | 0.7506 | -2 |
| TBBS | 0.5405 | 0.7528 | 0 |
| MBT | 0.5262 | 0.7631 | +2 |
| CBS | 0.5090 | 0.7294 | 0 |
| MBTS | 0.4971 | 0.7104 | 0 |
| DCBS | 0.4704 | 0.6860 | 0 |
| TMTD | 0.4302 | 0.6664 | 0 |
| DPG | 0.4052 | 0.6530 | 0 |
| AO-1010 | 0 | 0.2430 | 0 |

Note: RI_mul and RI_aws are the Risk Index from multiplicative coupling and additive weighted-sum coupling, respectively. *RI*_aws,i_ = 0.5*ToxPi*_i_ + 0.5*Exposure*_normalized,i_; ΔRank = Rank_aws_ − Rank_mul_; 0 indicates no change, (+) a lower rank, and (−) a higher rank under the additive weighted-sum coupling.

**Fig. S10.** ToxPi-based hazard prioritization results under two endpoint integration strategies and the corresponding dimension-level weight allocation. The blue curve represents ToxPi scores derived using the best-model approach, and the red curve represents ToxPi scores derived using the mean-model approach. The pie chart in the upper right illustrates the ToxPi slice weights. The four primary dimensions, persistence (P), bioaccumulation (B), mobility (M), and toxicity (T), were assigned equal weights of 25% each. Within each primary dimension, slices correspond to the specific endpoints included in that dimension, such as BIOWIN 2, BIOWIN 3, and BIOWIN 6, log BCF, log Koc, and multiple toxicity-related endpoints. Weights were allocated proportionally and equally among slices within the total weight assigned to each dimension.

**Table S6.** List of polycyclic aromatic hydrocarbons (PAHs).

| **Name** | **Source** | **Rank** | |
| --- | --- | --- | --- |
|  |  | **Average** | **Optimal** |
| Benzo[k]fluoranthene (BkFA) | '207-08-9 | 8 | 17 |
| 7,8-Benzfluoranthene (BjFA) | '205-82-3 | 9 | 15 |
| Indeno[1,2,3-cd]pyrene | '193-39-5 | 10 | 14 |
| Benzo[b]fluoranthene (BbFA) | '205-99-2 | 11 | 16 |
| Benzo[e]pyrene (BeP) | '192-97-2 | 23 | 36 |
| Benzo[a]pyrene (BaP) | '50-32-8 | 29 | 28 |
| Benz(a)anthracene (BaA) | '56-55-3 | 36 | 35 |
| Perylene | '198-55-0 | 37 | 38 |
| Pyrene | '129-00-0 | 44 | 43 |
| Chrysene (CHR) | '218-01-9 | 45 | 42 |
| Phenanthrene | '85-01-8 | 90 | 90 |
| Anthracene | '120-12-7 | 95 | 86 |
| Naphthalene | '91-20-3 | 244 | 239 |

Table S7. Intentional additives selected from the 82 high-priority chemicals for future concentration characterization in rubber formulations

| **Functional category** | **CAS No.** | **Abbreviation** |
| --- | --- | --- |
| Processing aid | 133-49-3 | PCTP |
| Antioxidant / antiozonant | 93-46-9 | — |
|  | 27417-40-9 | — |
|  | 74-31-7 | DPPD |
|  | 101-87-1 | — |
|  | 3081-14-9 | — |
|  | 90-30-2 | PANA |
|  | 10081-67-1 | — |
|  | 119-47-1 | AO-2246 |
|  | 4130-42-1 | — |
|  | 88-27-7 | — |
| UV / light stabilizer | 3846-71-7 | UV-320 |
|  | 3864-99-1 | UV-327 |
|  | 25973-55-1 | UV-328 |
|  | 36437-37-3 | UV-350 |
|  | 3147-75-9 | UV-329 |
|  | 3896-11-5 | UV-326 |
|  | 65447-77-0 | — |
| Flame retardant / plasticizer | 1163-19-5 | DBDPO / DecaBDE |
|  | 79-94-7 | TBBPA |
|  | 3194-55-6 | HBCD |
|  | 134237-52-8 | γ-HBCD |
|  | 32588-76-4 | EBTPI |
|  | 85535-84-8 | SCCPs |
|  | 85535-85-9 | MCCPs |
|  | 13674-84-5 | TCPP |
|  | 115-96-8 | TCEP |
